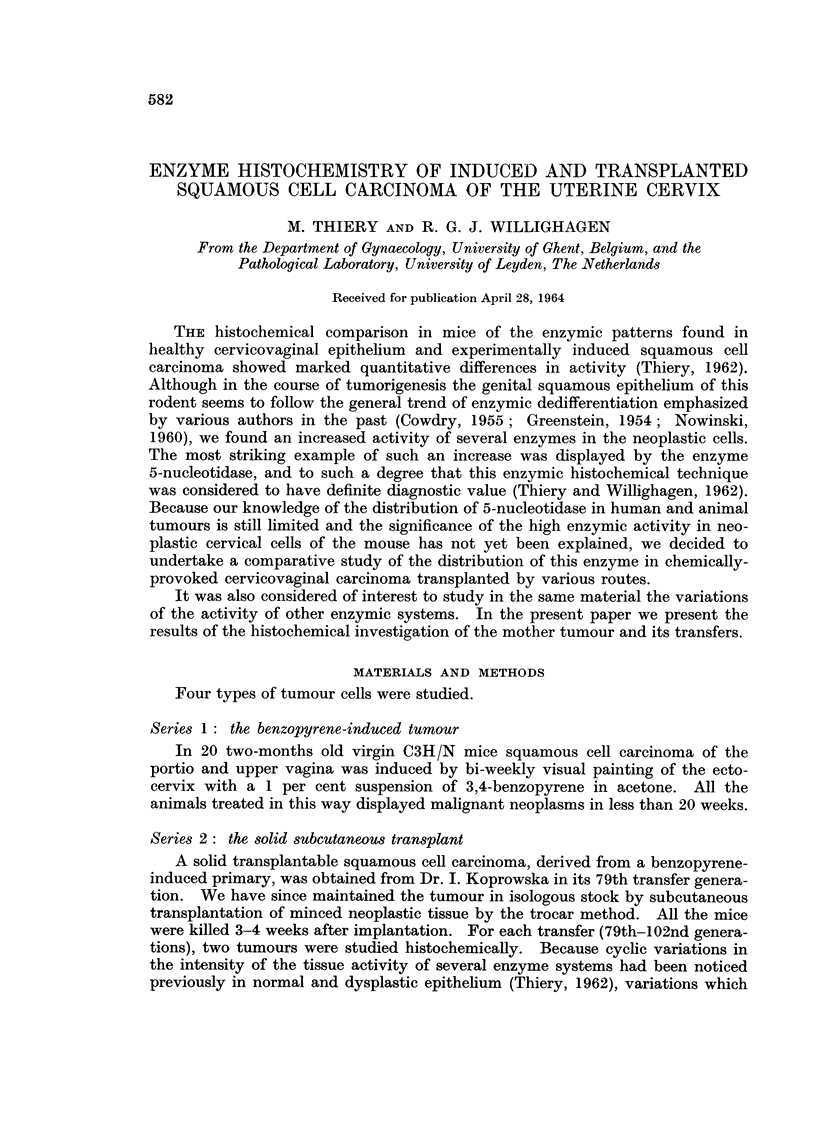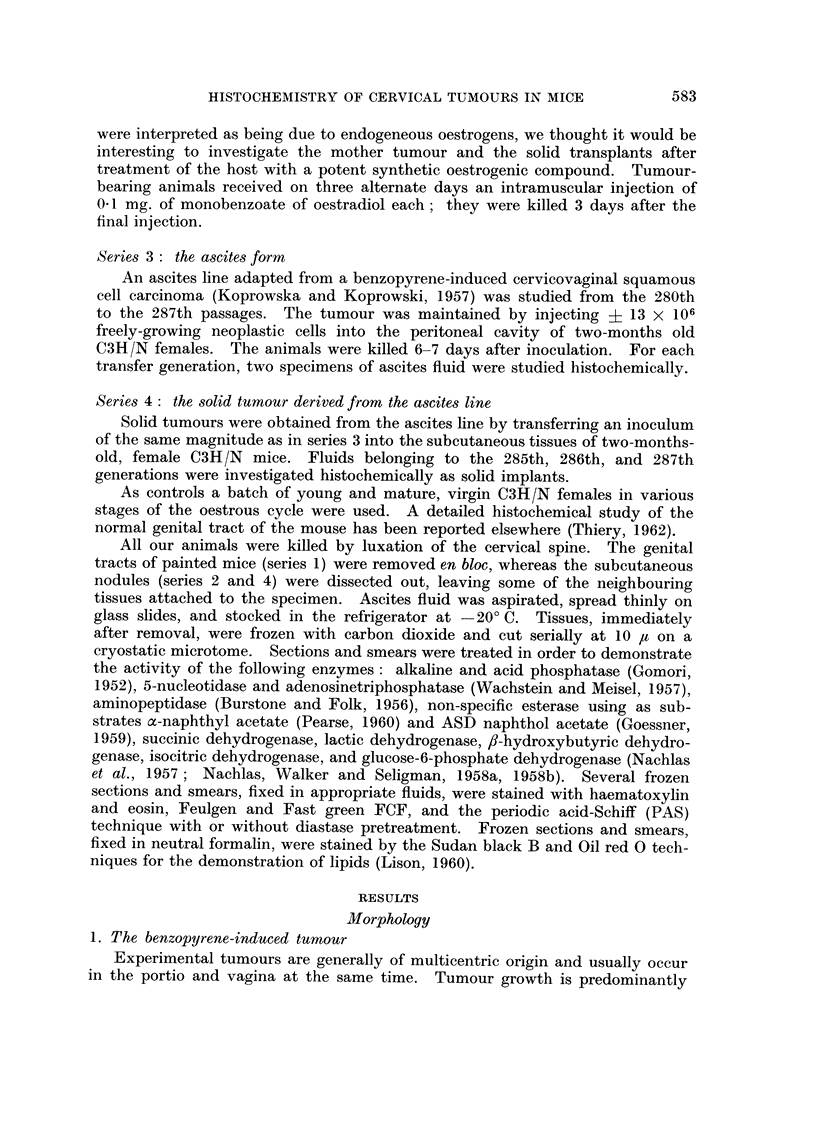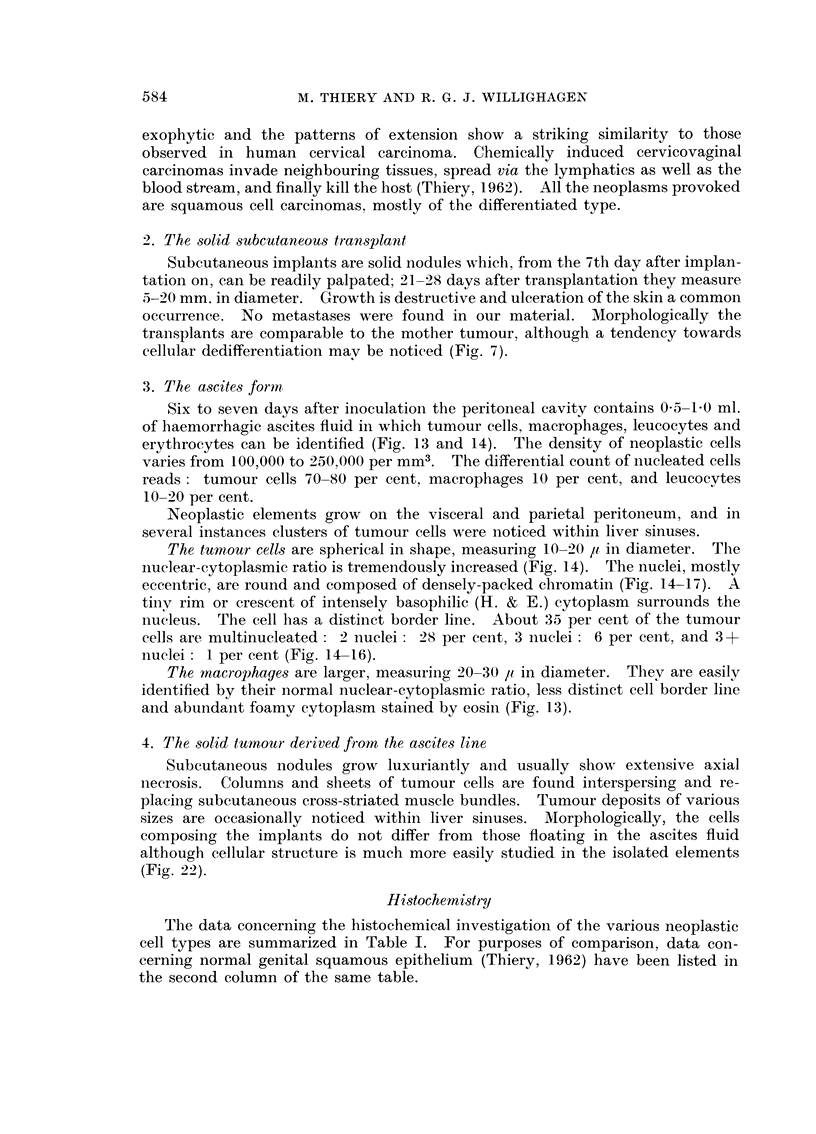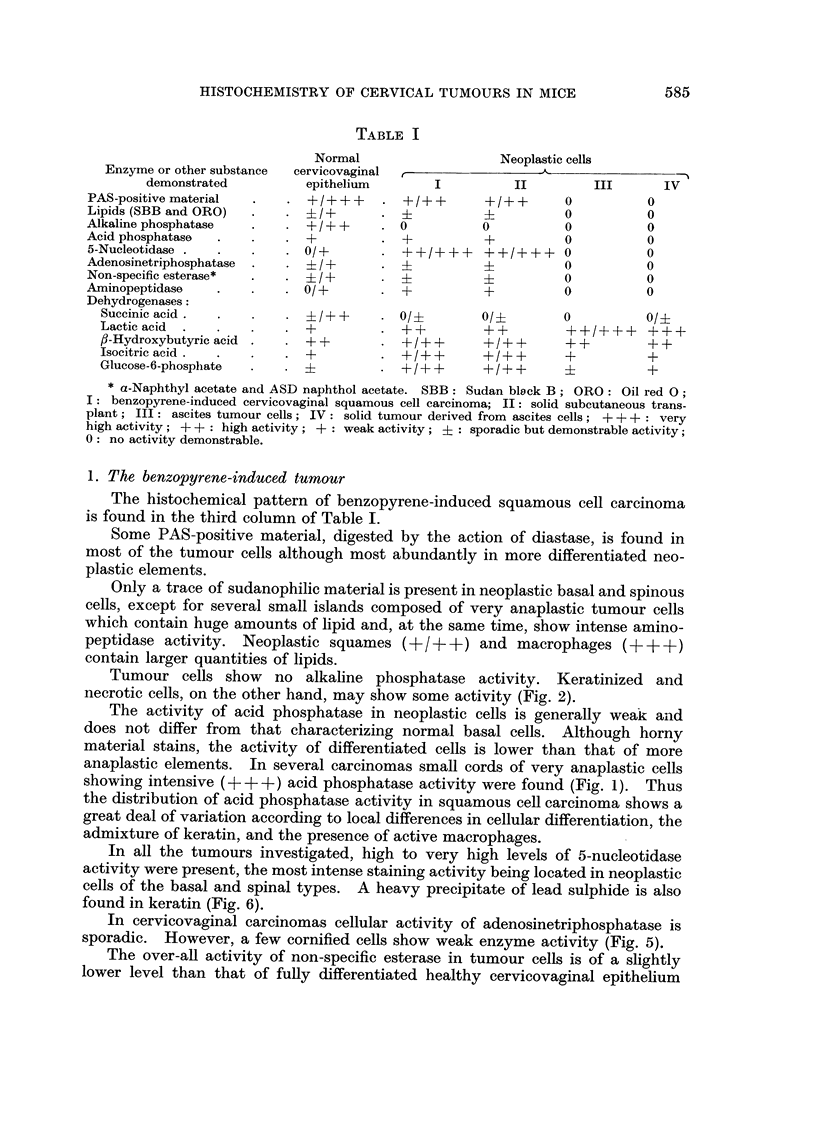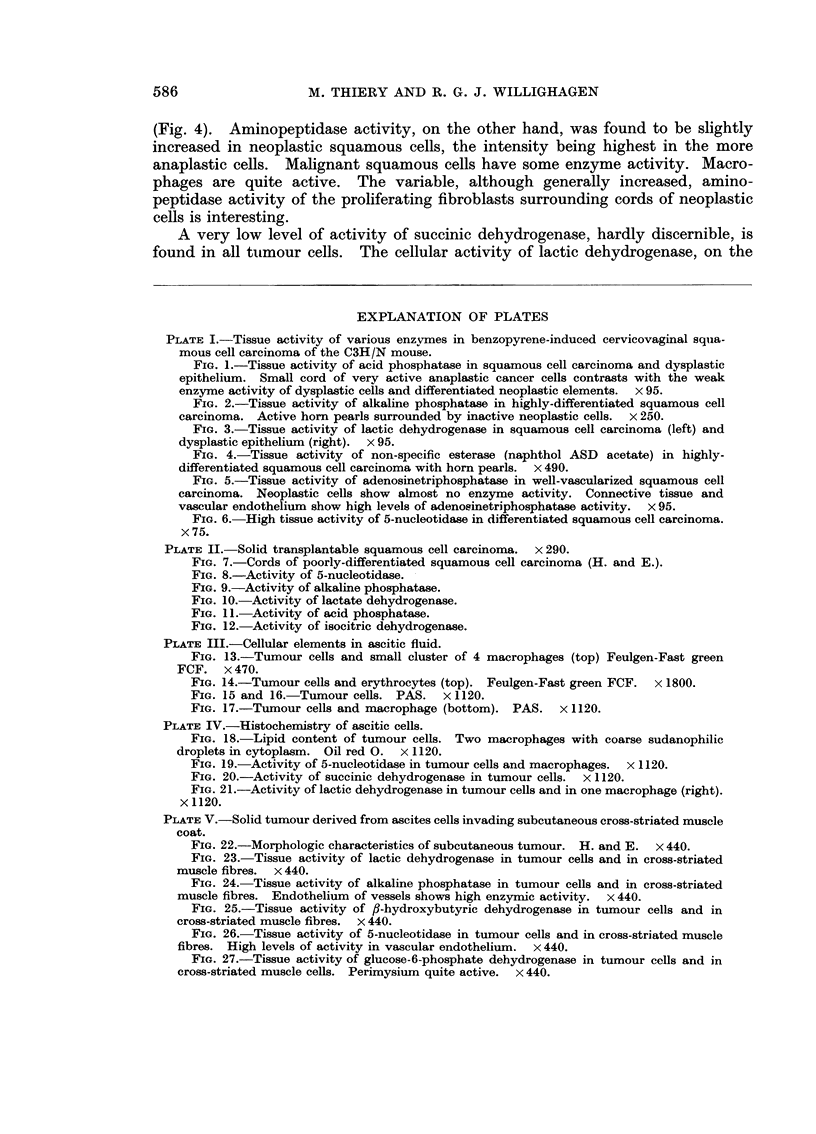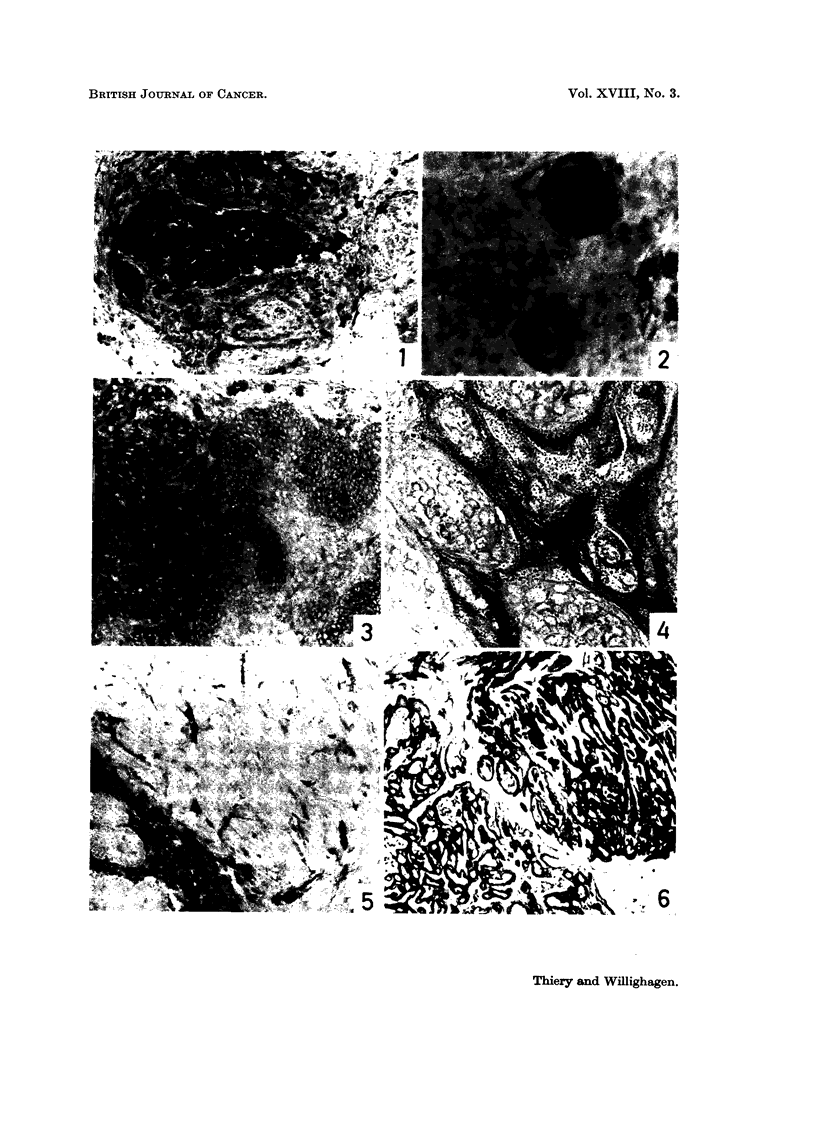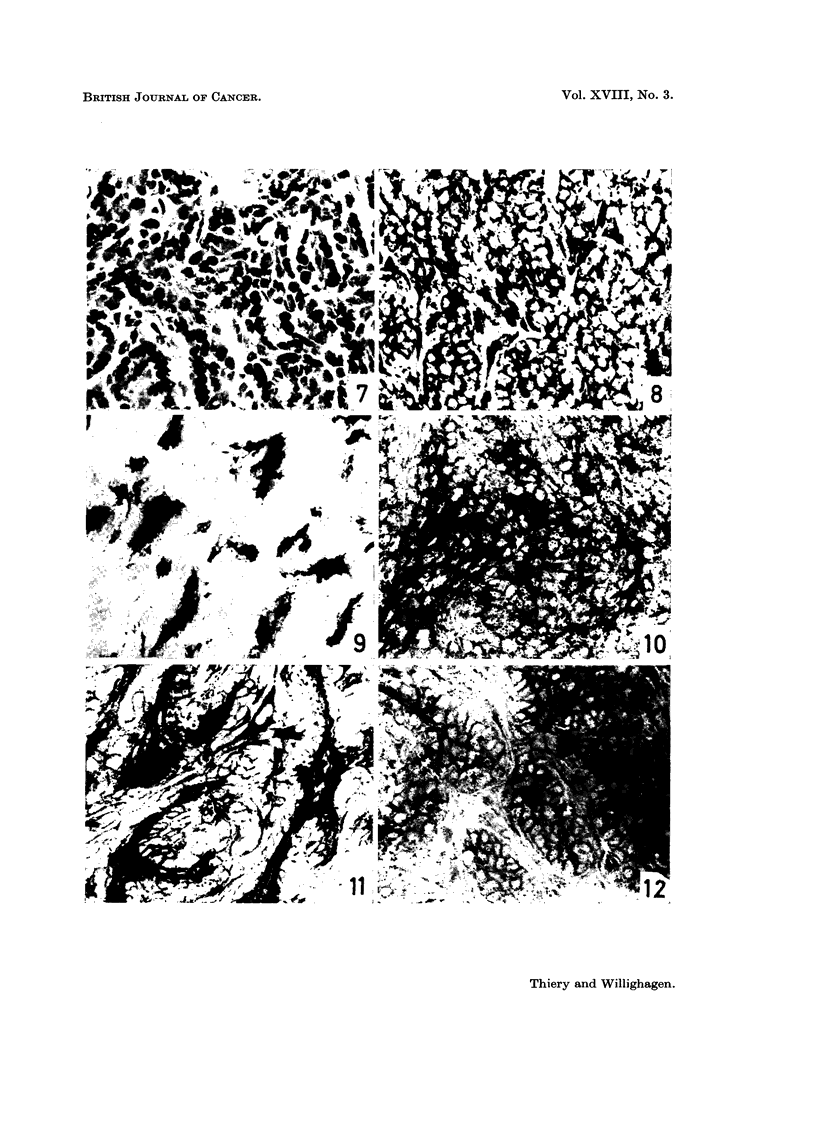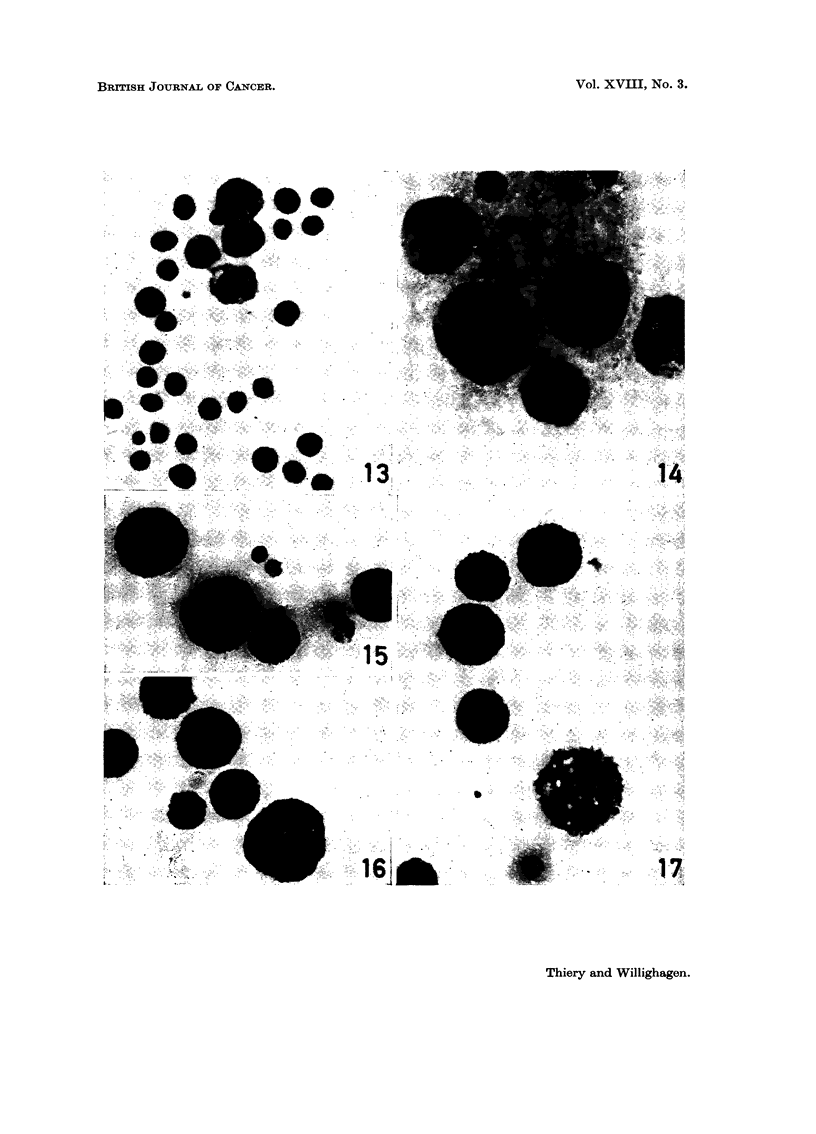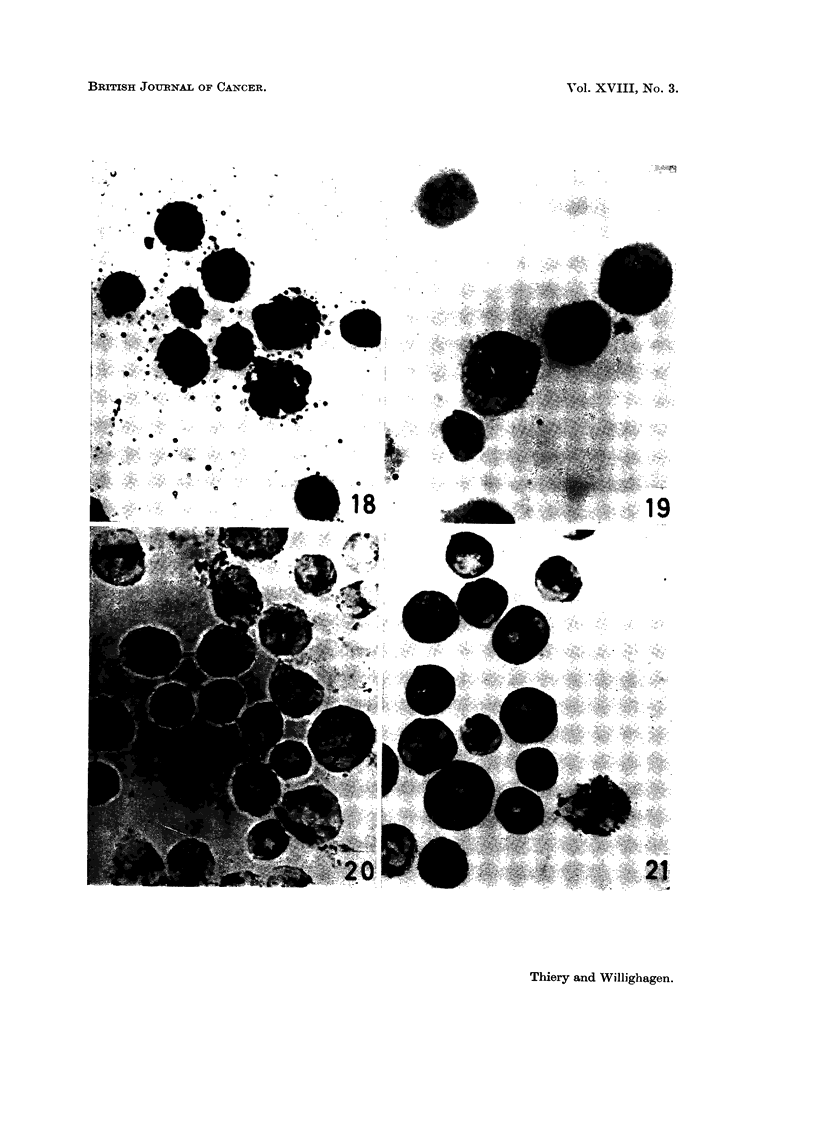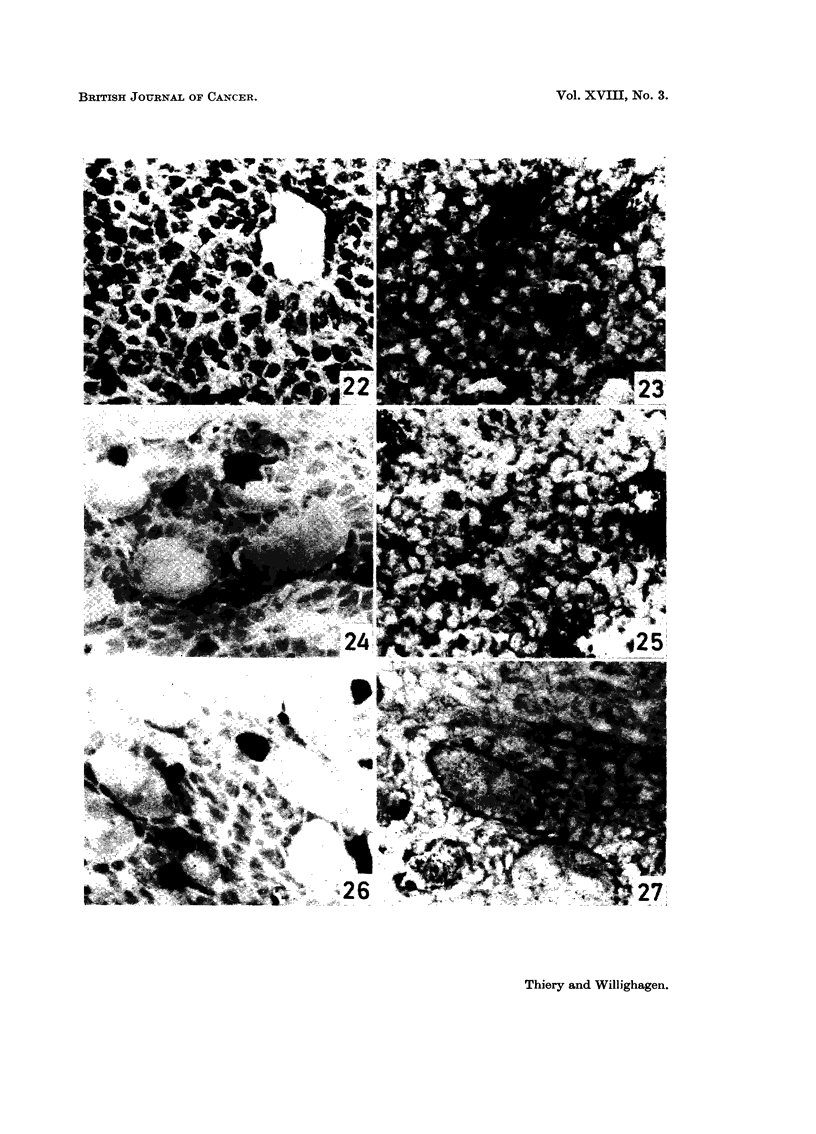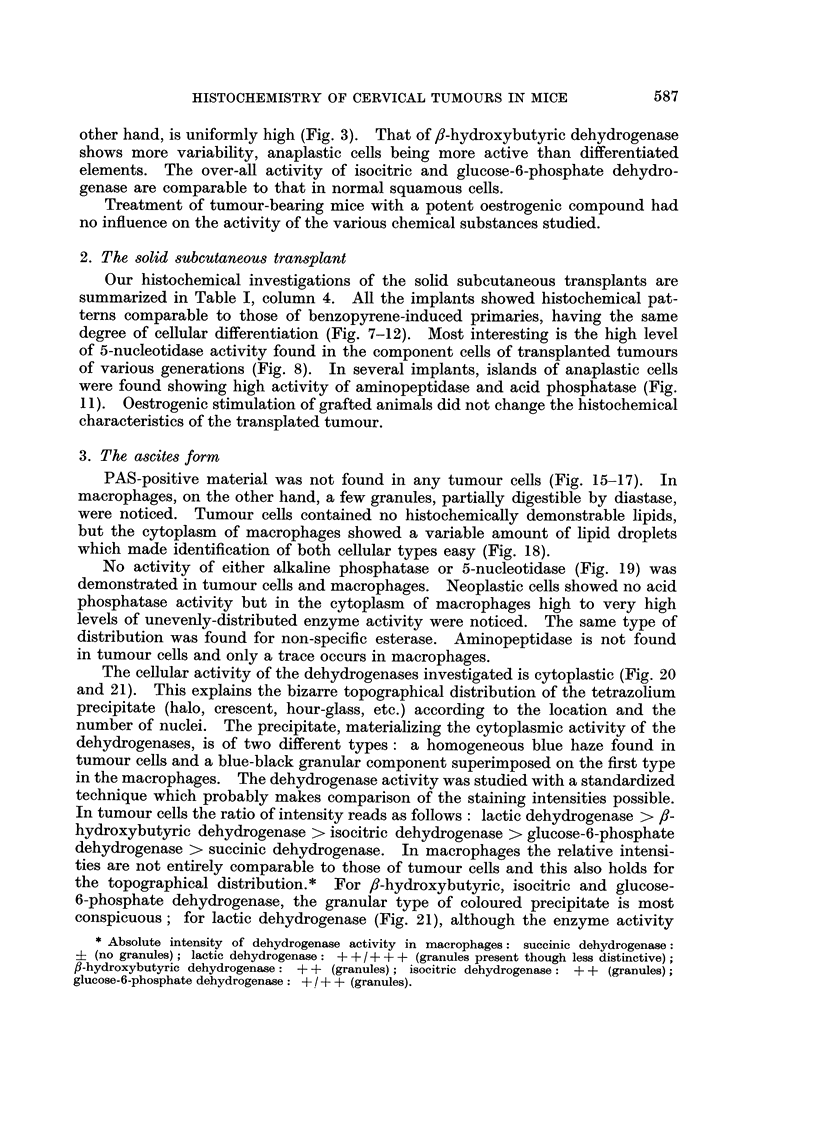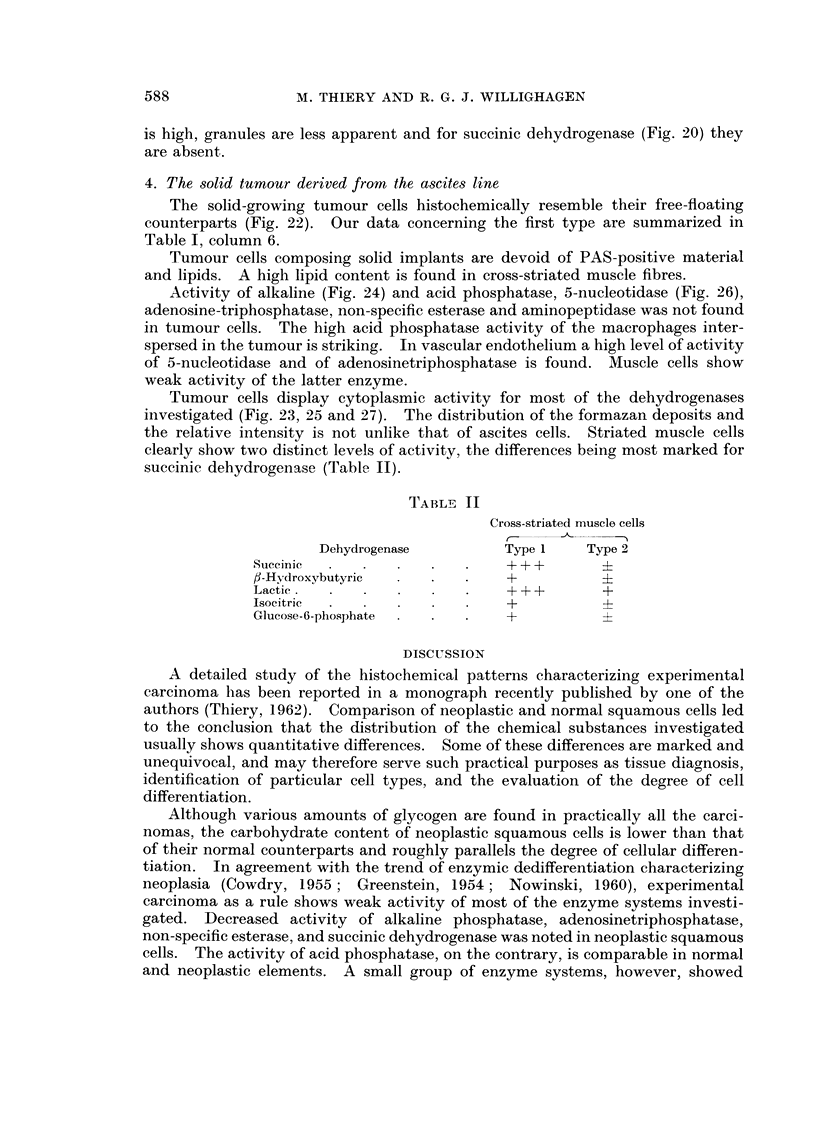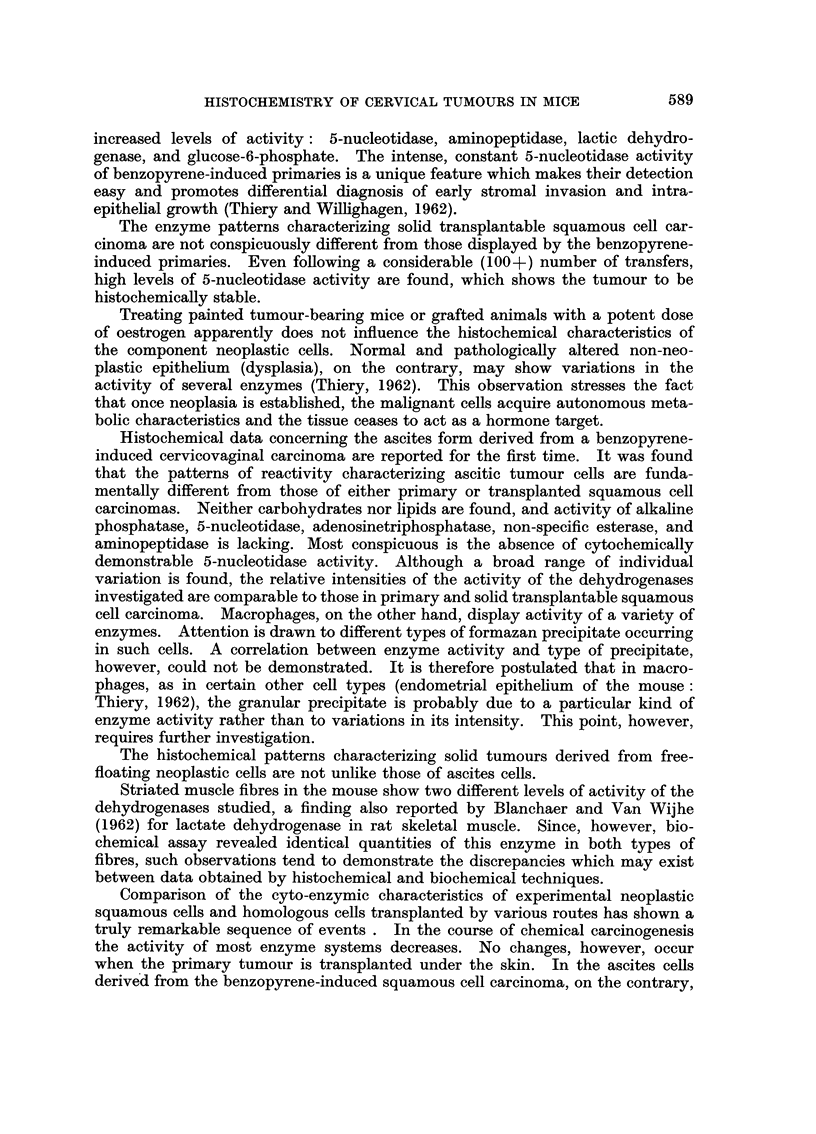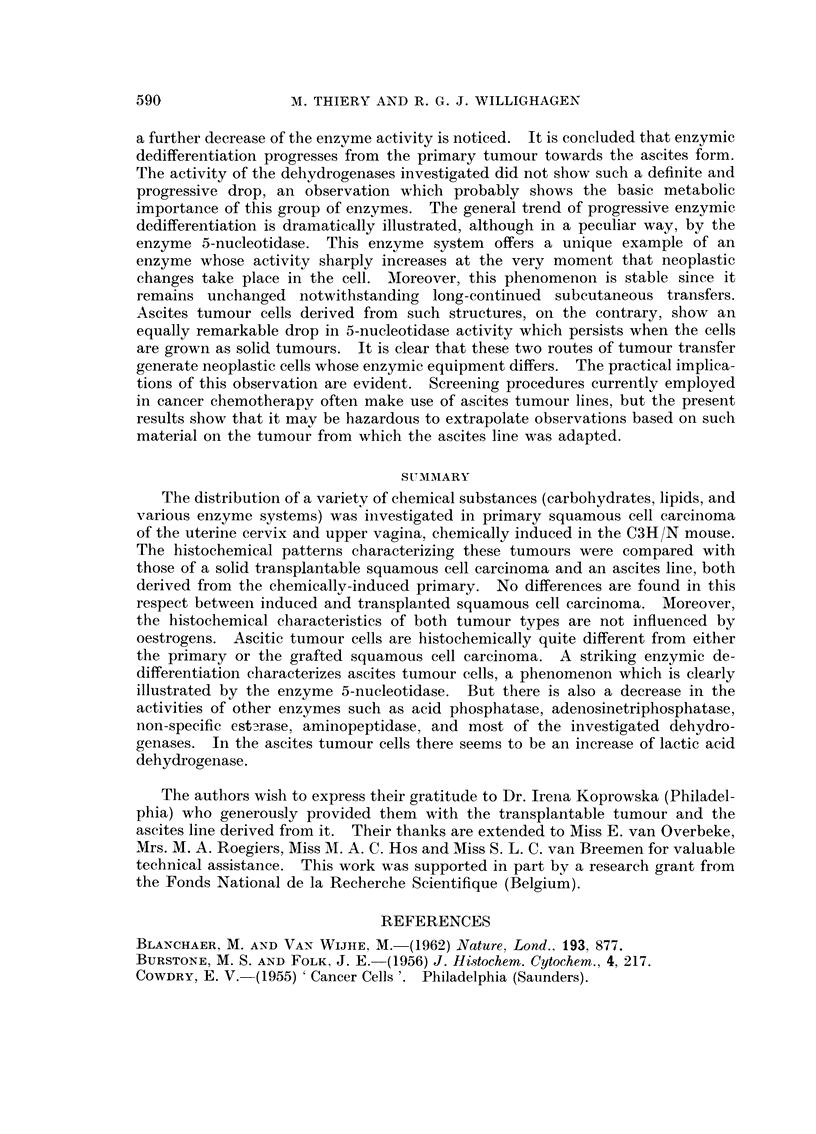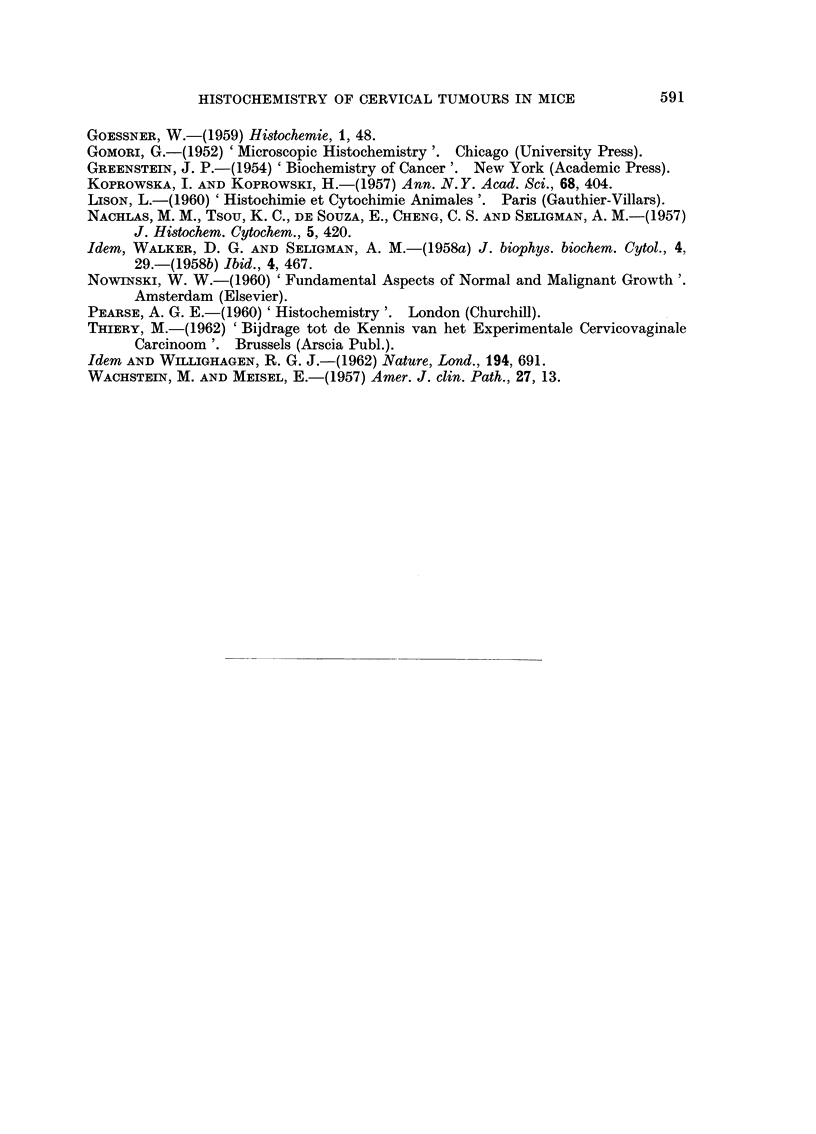# Enzyme Histochemistry of Induced and Transplanted Squamous Cell Carcinoma of the Uterine Cervix

**DOI:** 10.1038/bjc.1964.67

**Published:** 1964-09

**Authors:** M. Thiery, R. G. J. Willighagen

## Abstract

**Images:**


### ENZYME HISTOCHEMISTRY OF INDUCED AND TRANSPLANTED SQUAMOUS CELL CARCINOMA OF THE UTERINE CERVIX

M. THIERY AND R. G. J. WILLIGHAGEN

*From the Department of Gynaecology, University of Ghent, Belgium, and the Pathological Laboratory, University of Leyden, The Netherlands*

Received for publication April 28, 1964

THE histochemical comparison in mice of the enzymic patterns found in healthy cervicovaginal epithelium and experimentally induced squamous cell carcinoma showed marked quantitative differences in activity (Thiery, 1962). Although in the course of tumorigenesis the genital squamous epithelium of this rodent seems to follow the general trend of enzymic dedifferentiation emphasized by various authors in the past (Cowdry, 1955 ; Greenstein, 1954 ; Nowinski, 1960), we found an increased activity of several enzymes in the neoplastic cells. The most striking example of such an increase was displayed by the enzyme 5-nucleotidase, and to such a degree that this enzymic histochemical technique was considered to have definite diagnostic value (Thiery and Willighagen, 1962). Because our knowledge of the distribution of 5-nucleotidase in human and animal tumours is still limited and the significance of the high enzymic activity in neoplastic cervical cells of the mouse has not yet been explained, we decided to undertake a comparative study of the distribution of this enzyme in chemically-provoked cervicovaginal carcinoma transplanted by various routes.

It was also considered of interest to study in the same material the variations of the activity of other enzymic systems. In the present paper we present the results of the histochemical investigation of the mother tumour and its transfers.

### MATERIALS AND METHODS

Four types of tumour cells were studied.

### *Series* 1 : *the benzopyrene-induced tumour*

In 20 two-months old virgin C3H/N mice squamous cell carcinoma of the portio and upper vagina was induced by bi-weekly visual painting of the ecto-cervix with a 1 per cent suspension of 3,4-benzopyrene in acetone. All the animals treated in this way displayed malignant neoplasms in less than 20 weeks.

### *Series* 2 : *the solid subcutaneous transplant*

A solid transplantable squamous cell carcinoma, derived from a benzopyrene-induced primary, was obtained from Dr. I. Koprowska in its 79th transfer generation. We have since maintained the tumour in isologous stock by subcutaneous transplantation of minced neoplastic tissue by the trocar method. All the mice were killed 3–4 weeks after implantation. For each transfer (79th–102nd generations), two tumours were studied histochemically. Because cyclic variations in the intensity of the tissue activity of several enzyme systems had been noticed previously in normal and dysplastic epithelium (Thiery, 1962), variations which were interpreted as being due to endogeneous oestrogens, we thought it would be interesting to investigate the mother tumour and the solid transplants after treatment of the host with a potent synthetic oestrogenic compound. Tumour-bearing animals received on three alternate days an intramuscular injection of 0·1 mg. of monobenzoate of oestradiol each; they were killed 3 days after the final injection.

*Series* 3 : *the ascites form*

An ascites line adapted from a benzopyrene-induced cervicovaginal squamous cell carcinoma (Koprowska and Koprowski, 1957) was studied from the 280th to the 287th passages. The tumour was maintained by injecting $\pm 13 \times 10^6$ freely-growing neoplastic cells into the peritoneal cavity of two-months old C3H/N females. The animals were killed 6–7 days after inoculation. For each transfer generation, two specimens of ascites fluid were studied histochemically.

*Series* 4 : *the solid tumour derived from the ascites line*

Solid tumours were obtained from the ascites line by transferring an inoculum of the same magnitude as in series 3 into the subcutaneous tissues of two-months-old, female C3H/N mice. Fluids belonging to the 285th, 286th, and 287th generations were investigated histochemically as solid implants.

As controls a batch of young and mature, virgin C3H/N females in various stages of the oestrous cycle were used. A detailed histochemical study of the normal genital tract of the mouse has been reported elsewhere (Thiery, 1962).

All our animals were killed by luxation of the cervical spine. The genital tracts of painted mice (series 1) were removed *en bloc*, whereas the subcutaneous nodules (series 2 and 4) were dissected out, leaving some of the neighbouring tissues attached to the specimen. Ascites fluid was aspirated, spread thinly on glass slides, and stocked in the refrigerator at $-20°$ C. Tissues, immediately after removal, were frozen with carbon dioxide and cut serially at 10 $\mu$ on a cryostatic microtome. Sections and smears were treated in order to demonstrate the activity of the following enzymes: alkaline and acid phosphatase (Gomori, 1952), 5-nucleotidase and adenosinetriphosphatase (Wachstein and Meisel, 1957), aminopeptidase (Burstone and Folk, 1956), non-specific esterase using as substrates $\alpha$-naphthyl acetate (Pearse, 1960) and ASD naphthol acetate (Goessner, 1959), succinic dehydrogenase, lactic dehydrogenase, $\beta$-hydroxybutyric dehydrogenase, isocitric dehydrogenase, and glucose-6-phosphate dehydrogenase (Nachlas *et al.*, 1957; Nachlas, Walker and Seligman, 1958a, 1958b). Several frozen sections and smears, fixed in appropriate fluids, were stained with haematoxylin and eosin, Feulgen and Fast green FCF, and the periodic acid-Schiff (PAS) technique with or without diastase pretreatment. Frozen sections and smears, fixed in neutral formalin, were stained by the Sudan black B and Oil red O techniques for the demonstration of lipids (Lison, 1960).

### RESULTS

### *Morphology*

1. *The benzopyrene-induced tumour*

Experimental tumours are generally of multicentric origin and usually occur in the portio and vagina at the same time. Tumour growth is predominantly exophytic and the patterns of extension show a striking similarity to those observed in human cervical carcinoma. Chemically induced cervicovaginal carcinomas invade neighbouring tissues, spread *via* the lymphatics as well as the blood stream, and finally kill the host (Thiery, 1962). All the neoplasms provoked are squamous cell carcinomas, mostly of the differentiated type.

2. *The solid subcutaneous transplant*

Subcutaneous implants are solid nodules which, from the 7th day after implantation on, can be readily palpated; 21–28 days after transplantation they measure 5–20 mm. in diameter. Growth is destructive and ulceration of the skin a common occurrence. No metastases were found in our material. Morphologically the transplants are comparable to the mother tumour, although a tendency towards cellular dedifferentiation may be noticed (Fig. 7).

3. *The ascites form*

Six to seven days after inoculation the peritoneal cavity contains 0·5–1·0 ml. of haemorrhagic ascites fluid in which tumour cells, macrophages, leucocytes and erythrocytes can be identified (Fig. 13 and 14). The density of neoplastic cells varies from 100,000 to 250,000 per $mm^3$. The differential count of nucleated cells reads: tumour cells 70–80 per cent, macrophages 10 per cent, and leucocytes 10–20 per cent.

Neoplastic elements grow on the visceral and parietal peritoneum, and in several instances clusters of tumour cells were noticed within liver sinuses.

*The tumour cells* are spherical in shape, measuring 10–20 $\mu$ in diameter. The nuclear-cytoplasmic ratio is tremendously increased (Fig. 14). The nuclei, mostly eccentric, are round and composed of densely-packed chromatin (Fig. 14–17). A tiny rim or crescent of intensely basophilic (H. & E.) cytoplasm surrounds the nucleus. The cell has a distinct border line. About 35 per cent of the tumour cells are multinucleated: 2 nuclei: 28 per cent, 3 nuclei: 6 per cent, and 3+ nuclei: 1 per cent (Fig. 14–16).

*The macrophages* are larger, measuring 20–30 $\mu$ in diameter. They are easily identified by their normal nuclear-cytoplasmic ratio, less distinct cell border line and abundant foamy cytoplasm stained by eosin (Fig. 13).

4. *The solid tumour derived from the ascites line*

Subcutaneous nodules grow luxuriantly and usually show extensive axial necrosis. Columns and sheets of tumour cells are found interspersing and replacing subcutaneous cross-striated muscle bundles. Tumour deposits of various sizes are occasionally noticed within liver sinuses. Morphologically, the cells composing the implants do not differ from those floating in the ascites fluid although cellular structure is much more easily studied in the isolated elements (Fig. 22).

### *Histochemistry*

The data concerning the histochemical investigation of the various neoplastic cell types are summarized in Table I. For purposes of comparison, data concerning normal genital squamous epithelium (Thiery, 1962) have been listed in the second column of the same table.

TABLE I

| Enzyme or other substance demonstrated | Normal cervicovaginal epithelium | Neoplastic cells I | II | III | IV |
|---|---|---|---|---|---|
| PAS-positive material | +/+++ | +/++ | +/++ | 0 | 0 |
| Lipids (SBB and ORO) | ±/+ | ± | ± | 0 | 0 |
| Alkaline phosphatase | +/++ | 0 | 0 | 0 | 0 |
| Acid phosphatase | + | + | + | 0 | 0 |
| 5-Nucleotidase | 0/+ | ++/+++ | ++/+++ | 0 | 0 |
| Adenosinetriphosphatase | ±/+ | ± | ± | 0 | 0 |
| Non-specific esterase* | ±/+ | ± | ± | 0 | 0 |
| Aminopeptidase | 0/+ | + | + | 0 | 0 |
| Dehydrogenases: | | | | | |
| Succinic acid | ±/++ | 0/± | 0/± | 0 | 0/± |
| Lactic acid | + | ++ | ++ | ++/+++ | +++ |
| β-Hydroxybutyric acid | ++ | +/++ | +/++ | ++ | ++ |
| Isocitric acid | + | +/++ | +/++ | + | + |
| Glucose-6-phosphate | ± | +/++ | +/++ | ± | + |

* α-Naphthyl acetate and ASD naphthol acetate. SBB : Sudan black B ; ORO : Oil red O ; I : benzopyrene-induced cervicovaginal squamous cell carcinoma; II : solid subcutaneous transplant ; III : ascites tumour cells ; IV : solid tumour derived from ascites cells ; +++ : very high activity ; ++ : high activity ; + : weak activity ; ± : sporadic but demonstrable activity ; 0 : no activity demonstrable.

1. *The benzopyrene-induced tumour*

The histochemical pattern of benzopyrene-induced squamous cell carcinoma is found in the third column of Table I.

Some PAS-positive material, digested by the action of diastase, is found in most of the tumour cells although most abundantly in more differentiated neoplastic elements.

Only a trace of sudanophilic material is present in neoplastic basal and spinous cells, except for several small islands composed of very anaplastic tumour cells which contain huge amounts of lipid and, at the same time, show intense aminopeptidase activity. Neoplastic squames (+/++) and macrophages (+++) contain larger quantities of lipids.

Tumour cells show no alkaline phosphatase activity. Keratinized and necrotic cells, on the other hand, may show some activity (Fig. 2).

The activity of acid phosphatase in neoplastic cells is generally weak and does not differ from that characterizing normal basal cells. Although horny material stains, the activity of differentiated cells is lower than that of more anaplastic elements. In several carcinomas small cords of very anaplastic cells showing intensive (+++) acid phosphatase activity were found (Fig. 1). Thus the distribution of acid phosphatase activity in squamous cell carcinoma shows a great deal of variation according to local differences in cellular differentiation, the admixture of keratin, and the presence of active macrophages.

In all the tumours investigated, high to very high levels of 5-nucleotidase activity were present, the most intense staining activity being located in neoplastic cells of the basal and spinal types. A heavy precipitate of lead sulphide is also found in keratin (Fig. 6).

In cervicovaginal carcinomas cellular activity of adenosinetriphosphatase is sporadic. However, a few cornified cells show weak enzyme activity (Fig. 5).

The over-all activity of non-specific esterase in tumour cells is of a slightly lower level than that of fully differentiated healthy cervicovaginal epithelium

(Fig. 4). Aminopeptidase activity, on the other hand, was found to be slightly increased in neoplastic squamous cells, the intensity being highest in the more anaplastic cells. Malignant squamous cells have some enzyme activity. Macrophages are quite active. The variable, although generally increased, aminopeptidase activity of the proliferating fibroblasts surrounding cords of neoplastic cells is interesting.

A very low level of activity of succinic dehydrogenase, hardly discernible, is found in all tumour cells. The cellular activity of lactic dehydrogenase, on the

---

### EXPLANATION OF PLATES

PLATE I.—Tissue activity of various enzymes in benzopyrene-induced cervicovaginal squamous cell carcinoma of the C3H/N mouse.

FIG. 1.—Tissue activity of acid phosphatase in squamous cell carcinoma and dysplastic epithelium. Small cord of very active anaplastic cancer cells contrasts with the weak enzyme activity of dysplastic cells and differentiated neoplastic elements. × 95.

FIG. 2.—Tissue activity of alkaline phosphatase in highly-differentiated squamous cell carcinoma. Active horn pearls surrounded by inactive neoplastic cells. × 250.

FIG. 3.—Tissue activity of lactic dehydrogenase in squamous cell carcinoma (left) and dysplastic epithelium (right). × 95.

FIG. 4.—Tissue activity of non-specific esterase (naphthol ASD acetate) in highly-differentiated squamous cell carcinoma with horn pearls. × 490.

FIG. 5.—Tissue activity of adenosinetriphosphatase in well-vascularized squamous cell carcinoma. Neoplastic cells show almost no enzyme activity. Connective tissue and vascular endothelium show high levels of adenosinetriphosphatase activity. × 95.

FIG. 6.—High tissue activity of 5-nucleotidase in differentiated squamous cell carcinoma. × 75.

PLATE II.—Solid transplantable squamous cell carcinoma. × 290.

FIG. 7.—Cords of poorly-differentiated squamous cell carcinoma (H. and E.).

FIG. 8.—Activity of 5-nucleotidase.

FIG. 9.—Activity of alkaline phosphatase.

FIG. 10.—Activity of lactate dehydrogenase.

FIG. 11.—Activity of acid phosphatase.

FIG. 12.—Activity of isocitric dehydrogenase.

PLATE III.—Cellular elements in ascitic fluid.

FIG. 13.—Tumour cells and small cluster of 4 macrophages (top) Feulgen-Fast green FCF. × 470.

FIG. 14.—Tumour cells and erythrocytes (top). Feulgen-Fast green FCF. × 1800.

FIG. 15 and 16.—Tumour cells. PAS. × 1120.

FIG. 17.—Tumour cells and macrophage (bottom). PAS. × 1120.

PLATE IV.—Histochemistry of ascitic cells.

FIG. 18.—Lipid content of tumour cells. Two macrophages with coarse sudanophilic droplets in cytoplasm. Oil red O. × 1120.

FIG. 19.—Activity of 5-nucleotidase in tumour cells and macrophages. × 1120.

FIG. 20.—Activity of succinic dehydrogenase in tumour cells. × 1120.

FIG. 21.—Activity of lactic dehydrogenase in tumour cells and in one macrophage (right). × 1120.

PLATE V.—Solid tumour derived from ascites cells invading subcutaneous cross-striated muscle coat.

FIG. 22.—Morphologic characteristics of subcutaneous tumour. H. and E. × 440.

FIG. 23.—Tissue activity of lactic dehydrogenase in tumour cells and in cross-striated muscle fibres. × 440.

FIG. 24.—Tissue activity of alkaline phosphatase in tumour cells and in cross-striated muscle fibres. Endothelium of vessels shows high enzymic activity. × 440.

FIG. 25.—Tissue activity of $\beta$-hydroxybutyric dehydrogenase in tumour cells and in cross-striated muscle fibres. × 440.

FIG. 26.—Tissue activity of 5-nucleotidase in tumour cells and in cross-striated muscle fibres. High levels of activity in vascular endothelium. × 440.

FIG. 27.—Tissue activity of glucose-6-phosphate dehydrogenase in tumour cells and in cross-striated muscle cells. Perimysium quite active. × 440.

Thiery and Willighagen.

Thiery and Willighagen.

Thiery and Willighagen.

Thiery and Willighagen.

Thiery and Willighagen.

other hand, is uniformly high (Fig. 3). That of $\beta$-hydroxybutyric dehydrogenase shows more variability, anaplastic cells being more active than differentiated elements. The over-all activity of isocitric and glucose-6-phosphate dehydrogenase are comparable to that in normal squamous cells.

Treatment of tumour-bearing mice with a potent oestrogenic compound had no influence on the activity of the various chemical substances studied.

2. *The solid subcutaneous transplant*

Our histochemical investigations of the solid subcutaneous transplants are summarized in Table I, column 4. All the implants showed histochemical patterns comparable to those of benzopyrene-induced primaries, having the same degree of cellular differentiation (Fig. 7–12). Most interesting is the high level of 5-nucleotidase activity found in the component cells of transplanted tumours of various generations (Fig. 8). In several implants, islands of anaplastic cells were found showing high activity of aminopeptidase and acid phosphatase (Fig. 11). Oestrogenic stimulation of grafted animals did not change the histochemical characteristics of the transplated tumour.

3. *The ascites form*

PAS-positive material was not found in any tumour cells (Fig. 15–17). In macrophages, on the other hand, a few granules, partially digestible by diastase, were noticed. Tumour cells contained no histochemically demonstrable lipids, but the cytoplasm of macrophages showed a variable amount of lipid droplets which made identification of both cellular types easy (Fig. 18).

No activity of either alkaline phosphatase or 5-nucleotidase (Fig. 19) was demonstrated in tumour cells and macrophages. Neoplastic cells showed no acid phosphatase activity but in the cytoplasm of macrophages high to very high levels of unevenly-distributed enzyme activity were noticed. The same type of distribution was found for non-specific esterase. Aminopeptidase is not found in tumour cells and only a trace occurs in macrophages.

The cellular activity of the dehydrogenases investigated is cytoplastic (Fig. 20 and 21). This explains the bizarre topographical distribution of the tetrazolium precipitate (halo, crescent, hour-glass, etc.) according to the location and the number of nuclei. The precipitate, materializing the cytoplasmic activity of the dehydrogenases, is of two different types : a homogeneous blue haze found in tumour cells and a blue-black granular component superimposed on the first type in the macrophages. The dehydrogenase activity was studied with a standardized technique which probably makes comparison of the staining intensities possible. In tumour cells the ratio of intensity reads as follows : lactic dehydrogenase $>$ $\beta$-hydroxybutyric dehydrogenase $>$ isocitric dehydrogenase $>$ glucose-6-phosphate dehydrogenase $>$ succinic dehydrogenase. In macrophages the relative intensities are not entirely comparable to those of tumour cells and this also holds for the topographical distribution.* For $\beta$-hydroxybutyric, isocitric and glucose-6-phosphate dehydrogenase, the granular type of coloured precipitate is most conspicuous ; for lactic dehydrogenase (Fig. 21), although the enzyme activity

* Absolute intensity of dehydrogenase activity in macrophages : succinic dehydrogenase : $\pm$ (no granules) ; lactic dehydrogenase : $++/+++$ (granules present though less distinctive) ; $\beta$-hydroxybutyric dehydrogenase : $++$ (granules) ; isocitric dehydrogenase : $++$ (granules) ; glucose-6-phosphate dehydrogenase : $+/++$ (granules).

is high, granules are less apparent and for succinic dehydrogenase (Fig. 20) they are absent.

4. *The solid tumour derived from the ascites line*

The solid-growing tumour cells histochemically resemble their free-floating counterparts (Fig. 22). Our data concerning the first type are summarized in Table I, column 6.

Tumour cells composing solid implants are devoid of PAS-positive material and lipids. A high lipid content is found in cross-striated muscle fibres.

Activity of alkaline (Fig. 24) and acid phosphatase, 5-nucleotidase (Fig. 26), adenosine-triphosphatase, non-specific esterase and aminopeptidase was not found in tumour cells. The high acid phosphatase activity of the macrophages interspersed in the tumour is striking. In vascular endothelium a high level of activity of 5-nucleotidase and of adenosinetriphosphatase is found. Muscle cells show weak activity of the latter enzyme.

Tumour cells display cytoplasmic activity for most of the dehydrogenases investigated (Fig. 23, 25 and 27). The distribution of the formazan deposits and the relative intensity is not unlike that of ascites cells. Striated muscle cells clearly show two distinct levels of activity, the differences being most marked for succinic dehydrogenase (Table II).

TABLE II

| Dehydrogenase | Cross-striated muscle cells: Type 1 | Cross-striated muscle cells: Type 2 |
|---|---|---|
| Succinic . . . . . | +++ | ± |
| $\beta$-Hydroxybutyric . . . | + | ± |
| Lactic . . . . . . | +++ | + |
| Isocitric . . . . . | + | ± |
| Glucose-6-phosphate . . . | + | ± |

DISCUSSION

A detailed study of the histochemical patterns characterizing experimental carcinoma has been reported in a monograph recently published by one of the authors (Thiery, 1962). Comparison of neoplastic and normal squamous cells led to the conclusion that the distribution of the chemical substances investigated usually shows quantitative differences. Some of these differences are marked and unequivocal, and may therefore serve such practical purposes as tissue diagnosis, identification of particular cell types, and the evaluation of the degree of cell differentiation.

Although various amounts of glycogen are found in practically all the carcinomas, the carbohydrate content of neoplastic squamous cells is lower than that of their normal counterparts and roughly parallels the degree of cellular differentiation. In agreement with the trend of enzymic dedifferentiation characterizing neoplasia (Cowdry, 1955 ; Greenstein, 1954 ; Nowinski, 1960), experimental carcinoma as a rule shows weak activity of most of the enzyme systems investigated. Decreased activity of alkaline phosphatase, adenosinetriphosphatase, non-specific esterase, and succinic dehydrogenase was noted in neoplastic squamous cells. The activity of acid phosphatase, on the contrary, is comparable in normal and neoplastic elements. A small group of enzyme systems, however, showed increased levels of activity: 5-nucleotidase, aminopeptidase, lactic dehydrogenase, and glucose-6-phosphate. The intense, constant 5-nucleotidase activity of benzopyrene-induced primaries is a unique feature which makes their detection easy and promotes differential diagnosis of early stromal invasion and intra-epithelial growth (Thiery and Willighagen, 1962).

The enzyme patterns characterizing solid transplantable squamous cell carcinoma are not conspicuously different from those displayed by the benzopyrene-induced primaries. Even following a considerable (100+) number of transfers, high levels of 5-nucleotidase activity are found, which shows the tumour to be histochemically stable.

Treating painted tumour-bearing mice or grafted animals with a potent dose of oestrogen apparently does not influence the histochemical characteristics of the component neoplastic cells. Normal and pathologically altered non-neoplastic epithelium (dysplasia), on the contrary, may show variations in the activity of several enzymes (Thiery, 1962). This observation stresses the fact that once neoplasia is established, the malignant cells acquire autonomous metabolic characteristics and the tissue ceases to act as a hormone target.

Histochemical data concerning the ascites form derived from a benzopyrene-induced cervicovaginal carcinoma are reported for the first time. It was found that the patterns of reactivity characterizing ascitic tumour cells are fundamentally different from those of either primary or transplanted squamous cell carcinomas. Neither carbohydrates nor lipids are found, and activity of alkaline phosphatase, 5-nucleotidase, adenosinetriphosphatase, non-specific esterase, and aminopeptidase is lacking. Most conspicuous is the absence of cytochemically demonstrable 5-nucleotidase activity. Although a broad range of individual variation is found, the relative intensities of the activity of the dehydrogenases investigated are comparable to those in primary and solid transplantable squamous cell carcinoma. Macrophages, on the other hand, display activity of a variety of enzymes. Attention is drawn to different types of formazan precipitate occurring in such cells. A correlation between enzyme activity and type of precipitate, however, could not be demonstrated. It is therefore postulated that in macrophages, as in certain other cell types (endometrial epithelium of the mouse: Thiery, 1962), the granular precipitate is probably due to a particular kind of enzyme activity rather than to variations in its intensity. This point, however, requires further investigation.

The histochemical patterns characterizing solid tumours derived from free-floating neoplastic cells are not unlike those of ascites cells.

Striated muscle fibres in the mouse show two different levels of activity of the dehydrogenases studied, a finding also reported by Blanchaer and Van Wijhe (1962) for lactate dehydrogenase in rat skeletal muscle. Since, however, biochemical assay revealed identical quantities of this enzyme in both types of fibres, such observations tend to demonstrate the discrepancies which may exist between data obtained by histochemical and biochemical techniques.

Comparison of the cyto-enzymic characteristics of experimental neoplastic squamous cells and homologous cells transplanted by various routes has shown a truly remarkable sequence of events . In the course of chemical carcinogenesis the activity of most enzyme systems decreases. No changes, however, occur when the primary tumour is transplanted under the skin. In the ascites cells derived from the benzopyrene-induced squamous cell carcinoma, on the contrary, a further decrease of the enzyme activity is noticed. It is concluded that enzymic dedifferentiation progresses from the primary tumour towards the ascites form. The activity of the dehydrogenases investigated did not show such a definite and progressive drop, an observation which probably shows the basic metabolic importance of this group of enzymes. The general trend of progressive enzymic dedifferentiation is dramatically illustrated, although in a peculiar way, by the enzyme 5-nucleotidase. This enzyme system offers a unique example of an enzyme whose activity sharply increases at the very moment that neoplastic changes take place in the cell. Moreover, this phenomenon is stable since it remains unchanged notwithstanding long-continued subcutaneous transfers. Ascites tumour cells derived from such structures, on the contrary, show an equally remarkable drop in 5-nucleotidase activity which persists when the cells are grown as solid tumours. It is clear that these two routes of tumour transfer generate neoplastic cells whose enzymic equipment differs. The practical implications of this observation are evident. Screening procedures currently employed in cancer chemotherapy often make use of ascites tumour lines, but the present results show that it may be hazardous to extrapolate observations based on such material on the tumour from which the ascites line was adapted.

### SUMMARY

The distribution of a variety of chemical substances (carbohydrates, lipids, and various enzyme systems) was investigated in primary squamous cell carcinoma of the uterine cervix and upper vagina, chemically induced in the C3H/N mouse. The histochemical patterns characterizing these tumours were compared with those of a solid transplantable squamous cell carcinoma and an ascites line, both derived from the chemically-induced primary. No differences are found in this respect between induced and transplanted squamous cell carcinoma. Moreover, the histochemical characteristics of both tumour types are not influenced by oestrogens. Ascitic tumour cells are histochemically quite different from either the primary or the grafted squamous cell carcinoma. A striking enzymic dedifferentiation characterizes ascites tumour cells, a phenomenon which is clearly illustrated by the enzyme 5-nucleotidase. But there is also a decrease in the activities of other enzymes such as acid phosphatase, adenosinetriphosphatase, non-specific esterase, aminopeptidase, and most of the investigated dehydrogenases. In the ascites tumour cells there seems to be an increase of lactic acid dehydrogenase.

The authors wish to express their gratitude to Dr. Irena Koprowska (Philadelphia) who generously provided them with the transplantable tumour and the ascites line derived from it. Their thanks are extended to Miss E. van Overbeke, Mrs. M. A. Roegiers, Miss M. A. C. Hos and Miss S. L. C. van Breemen for valuable technical assistance. This work was supported in part by a research grant from the Fonds National de la Recherche Scientifique (Belgium).

### REFERENCES

BLANCHAER, M. AND VAN WIJHE, M.—(1962) *Nature, Lond.*, **193**, 877.
BURSTONE, M. S. AND FOLK, J. E.—(1956) *J. Histochem. Cytochem.*, **4**, 217.
COWDRY, E. V.—(1955) ‘ Cancer Cells ’. Philadelphia (Saunders).

GOESSNER, W.—(1959) *Histochemie*, **1**, 48.
GOMORI, G.—(1952) 'Microscopic Histochemistry'. Chicago (University Press).
GREENSTEIN, J. P.—(1954) 'Biochemistry of Cancer'. New York (Academic Press).
KOPROWSKA, I. AND KOPROWSKI, H.—(1957) *Ann. N.Y. Acad. Sci.*, **68**, 404.
LISON, L.—(1960) 'Histochimie et Cytochimie Animales'. Paris (Gauthier-Villars).
NACHLAS, M. M., TSOU, K. C., DE SOUZA, E., CHENG, C. S. AND SELIGMAN, A. M.—(1957) *J. Histochem. Cytochem.*, **5**, 420.
*Idem*, WALKER, D. G. AND SELIGMAN, A. M.—(1958*a*) *J. biophys. biochem. Cytol.*, **4**, 29.—(1958*b*) *Ibid.*, **4**, 467.
NOWINSKI, W. W.—(1960) 'Fundamental Aspects of Normal and Malignant Growth'. Amsterdam (Elsevier).
PEARSE, A. G. E.—(1960) 'Histochemistry'. London (Churchill).
THIERY, M.—(1962) 'Bijdrage tot de Kennis van het Experimentale Cervicovaginale Carcinoom'. Brussels (Arscia Publ.).
*Idem* AND WILLIGHAGEN, R. G. J.—(1962) *Nature, Lond.*, **194**, 691.
WACHSTEIN, M. AND MEISEL, E.—(1957) *Amer. J. clin. Path.*, **27**, 13.